# Sleep disorders and attention: a systematic review

**DOI:** 10.1590/0004-282X-ANP-2021-0182

**Published:** 2022-04-20

**Authors:** Thiago RODRIGUES, Nadia SHIGAEFF

**Affiliations:** 1 Universidade Federal de Juiz e Fora, Instituto de Ciências Humanas, Departamento de Psicologia, Juiz de Fora MG, Brazil.; 2 Universidade Federal de Juiz e Fora, Instituto de Ciências Humanas, Departamento de Psicologia, Núcleo Interdisciplinar de Pesquisa em Neuropsicologia e Gerontologia, Juiz de Fora MG, Brazil.

**Keywords:** Sleep, Sleep Wake Disorders, Attention, Sono, Transtornos do Sono-Vigília, Atenção

## Abstract

Background: Sleep is a special physiological state that occurs cyclically. The probable role of sleep in our organic functions remains to be explored to clarify the impact of sleep on brain functions. Sleep deprivation is known to affect all parts of the brain separately and independently, but further research is needed on the impact of sleep disorders on attention, particularly the specific types of attention that are most affected, and whether there is such a correlation. Objective: To conduct a systematic review of the possible correlation between sleep disorders and attentional performance. Methods: A systematic review and search at PubMed, SciELO, and Cochrane scientific databases for articles published in the last 10 years was carried out using the following keywords: sleep, attention, and attentional performance. Inclusion criteria were the use of attention tests and sleep disorders. Of the 1398 articles found, 15 were selected and included in this review. Results: The number of publications evaluating sleep and sleep disorders has increased, but is still limited. Of all the functions normally assessed, patients with sleep disorders perform worse on attention tasks, especially with sustained attention. However, these data require further investigation due to the complexity and diversity of the disorders, the small sample size of the included studies, and the fact that few studies used standardized tests. Conclusions: Our findings indicate that the correlation between sleep and attention is strong but limited. Few studies are devoted exclusively to the extent to which sleep disorders interferes with attention.

## RESUMO

Antecedentes: O sono é um estado fisiológico especial que ocorre de maneira cíclica. Sabe-se que a privação do sono afeta todas as partes do cérebro de forma separada e independente, mas sobre o impacto que os distúrbios do sono podem ter sobre a atenção ainda são necessárias mais pesquisas, especialmente sobre os tipos específicos de atenção mais afetados e se existe tal correlação. Objetivo: Conduzir uma revisão sistemática da possível correlação entre os distúrbios do sono e o desempenho atencional. Métodos: Revisão sistemática e busca nas bases de dados científicos PubMed, Scientific Electronic Library Online (SciELO) e Cochrane por publicações dos últimos 10 anos foram realizadas usando as seguintes palavras-chave: sono, atenção e desempenho atencional. Os critérios de inclusão foram o uso de testes de atenção e a abordagem de distúrbios do sono. Dos 1.398 artigos encontrados, 15 foram selecionados e incluídos nesta revisão. Resultados: O número de publicações que avaliavam sono e distúrbios do sono cresceu, mas ainda são escassos. De todas as funções normalmente avaliadas, os pacientes com distúrbios do sono têm pior desempenho nas tarefas de atenção, especialmente com atenção sustentada. No entanto, esses dados requerem investigações adicionais em virtude da complexidade e da diversidade dos distúrbios, do pequeno tamanho das amostras e dos poucos estudos que utilizaram testes padronizados. Conclusões: Nossas conclusões mostram que a correlação entre sono e atenção é forte mas limitada. Poucos estudos dedicam-se exclusivamente a descobrir até que ponto os distúrbios do sono atrapalham a atenção.

## INTRODUCTION

Sleep is a special physiological state that occurs cyclically in virtually all living things. Something very similar to sleep has been observed in a rudimentary rest and activity behavior (wake-sleep cycle) in animals as inferior on the zoological scale as insects^1^. Defining sleep is not a simple task, since some sleep stages show electrophysiological patterns similar to wakefulness when breathing patterns, eye movements, electroencephalogram (EEG), and some body movements are observed, which are completely different from other sleep stages where stillness and slow waves are observed in the EEG.

Although there have been an increasing number of studies on the subject recently, the probable role of sleep in our organic functioning has yet to be explored in order to clarify the impacts of this phenomenon on brain function. According to Eugene & Masiak, the neurobiological processes that occur during sleep are necessary for the maintenance of physical and cognitive health in - virtually - all species^2^. Sleep, the value of which is ignored by most people, also plays a fundamental role in maintaining higher cognitive functions that is not yet understood by science despite recent advances. In everyday life, sleep affects mood, memory, attention, sensory perception, and reasoning, that is, most of the cognitive aspects that connect people to their environment^3^. Sleep disorders can lead to poor quality of performance and sometimes have serious health consequences.

During the sleep cycle, some stages are responsible for rejuvenating and restoring the health of the brain. Generally, sleep is interpreted as an unconscious state, but brain activity continues to operate in its phases, and in a normal/healthy person, sleep is divided into two main phases: phases with non-rapid eye movements and phases with rapid eye movements (represented by NREM and REM, respectively). Recent studies show that the glymphatic system functions like a garbage disposal during NREM sleep, meticulously removing the byproducts of cellular waste^4^.

Sleep deprivation affects all parts of the brain separately and independently, and its effects are recurrent issues in patients with sleeplessness or insomnia. For example, the temporal lobe is primarily responsible for speech processing, and patients with insomnia often report slurred speech due to the brain’s inability to process neuronal signals at their best. In NREM sleep, histamine neurotransmitters are also turned off, so that their receptors become sensitive again^2^. Therefore, studies often find a correlation between anxiety and partial, or total, sleep deprivation.

The International Classification of Sleep Disorders (ICSD, 3rd edition) lists seven majors categories, while the four major sleep complaints include excessive daytime sleepiness, insomnia, abnormal movements or behaviors during sleep, and inability to fall asleep at the desired time^5^. Since a healthy sleep cycle keeps the brain and body healthy, the right conclusion to draw is that sleep disorders can significantly disrupt basic brain functions.

Attention is a topic that is widely discussed and studied in science and for which there are many conceptual definitions depending on the theoretical model, including psychology and neuroscience^6^.

In the present study, we start from the theoretical concept of neuropsychology that there are four types of ­attention: selective attention, sustained attention, divided attention, and alternate attention. In the context of sleep disorders, attention provides a comprehensive overview of its effects on cognition and the brain^7^.

Hennawy, who has discussed the influence of sleep disorders on Alzheimer’s disease (AD), develops a brief definition for the subtypes of attention used in this paper^7^. Selective attention is defined as the ability to focus on a single stimulus while blocking out all other distractions. Sustained attention, on the other hand, is defined as the ability to focus on a specific task for a long period of time. Divided attention is defined as the ability to attend to several tasks simultaneously. Finally, alternating attention or switching attention is the ability to effortlessly switch attention between tasks that require different cognitive demands.

The impact that sleep disorders may have on attention, particularly the specific types of attention that are most affected, and whether there is such a correlation is not well understood.

Considering the facts mentioned above, and taking into account the importance of studies investigating the association between sleep and attention for future public health research and the lack of studies summarizing the literature on this topic, the aim of this study was to conduct a systematic review that evaluates the relationship between sleep disorders and attentional performance.

## METHODS

The search was conducted between December 2020 and February 2021, following the guidelines of the Preferred Reporting Items for Systematic Reviews and Meta-Analyses (PRISMA). This systematic review was conducted in the SciELO, Medline/PubMed, and Cochrane databases within a 10-year period (2011-2020), using productions in both Portuguese and English. The following keywords were used in all databases: “attention and sleep” and “attentional performance and sleep”.

The inclusion criteria for this review were: productions from the past 10 years with samples of people aged 18 years or older with sleep disorders, cross-sectional or longitudinal scientific studies that used attentional tests with psychometric evidence of validity and reliability, and productions in Portuguese and/or English. Case reports, literature reviews, and systematic reviews were excluded.

A total of 1,682 references were found (1,327 in Cochrane, 321 in PubMED, and 34 in SciELO). Of these, 284 were duplicates and were therefore excluded. This left 1,398 references for abstract analysis. Sixty-one articles were selected and retrieved, and after reading the full-text, 15 were finally included (Table 1).

**Table 1.** General information about the 15 studies included in this review, according to the date of publication.

Study	Sample	Sleep disorder	Tests used	Main findings	Main limitations
Bawden et al.^17^	17 patients with polysomnographically diagnosed OSA in brief cognitive tests compared to 20 healthy controls (n=37)	OSA	MMSE, BCSB, DSS, ESS	OSA patients performed significantly worse than controls in the MMSE, in memory items from the BCSB, and in DS	Small sample
Waggoner et al.^12^	27 male and 2 female police officers (n=29)	Sleep deprivation	KSS, PVT, PSQI, ESS, MAPS	Significant lower scores on attention tests and psychomotor vigilance	Small sample, sleep disorder was self-reported
Moraes et al.^19^	38 participants divided into the following groups: Narcoleptic group (13 females; 6 males, mean age=37.58); control group (15 Female; 4 Male, mean age=34.42)	Narcolepsy	ESS-BR, VST, TMT	Narcoleptic patients showed higher degrees of EDS, an impaired executive attention at a temporal level and lower performance in working memory	Small sample
Karimi et al.^16^	72 males, 29 female drivers (n=101)	Hypersomnolence, insomnia, restless legs syndrome, OSA	ESS, KSS, FIS, ISI, IRLSS, Polysomnography, ANT, CTT, MWT	High prevalence of sleep disorders correlated with ANT poor results, improvement after OSA treatment	Few attention and cognitive tasks
Niu et al.^13^	62 nurses in a medical center in Taiwan and classified in two groups: fixed-day-shift group (control group, n=30) and rotating-shift group (experimental group, n=32)	Sleep deprivation	CPSQI, D2	Significant less selective attention, performance speed and accuracy on the attentional test were poorer in nurses working night shift than in those working day shift	Only woman participated, sleep deprivation was induced
Oliveira et al.^10^	Nursing staff in a hospital in Minas Gerais (n=102)	Sleep deprivation	DSS	Subjects in this study with nocturnal shift have impaired results in attention tests, and self-report less quality of sleep overall. Important to point out that these individuals also have greater total sleep hours in comparison.	No sleep tests, even self-reported scales. The correlation is uncertain and the sample is small.
Castronovo et al.^24^	17 patients with OSA and 15 controls (n=32)	OSA	MMSE, TMT, Stroop, ESS, PSQI	OSA patients showed impairments in most cognitive areas. After treatment, patients showed a significant improvement in sleepiness and in all cognitive tests, except for total time on Stroop test and Trial Making Test B	Small sample
Fortier-Brochu et al.^26^	25 adults with primary insomnia and 16 controls (n=25)	Insomnia	Polysomnographic recordings, spectral analysis of the electroencephalogram, ISI, PASAT, CPT-II	Individuals with insomnia had poorer overall performance compared to participants of the control group and committed more errors in attention and episodic memory tasks. Significant cognitive impairments were more frequent with insomnia.	Small Sample
Djonlagic et al.^14^	15 healthy controls and 29 patients with obstructive sleep apnea (n=44)	OSA	Polysomnography, PVT	Instant improvement in attention and vigilance based on PVT results	Small sample
Li et al.^11^	23 iRBD patients and 23 controls (n=46)	Idiopathic REM sleep behavior disorder (iRBD)	Polysomnography, MMSE, SCW, TMT, DSS	Negative correlation with the Trail Making B test, episodic verbal memory and non-verbal learning are the most affected domains in iRBD	Small sample
Luz et al.^18^	Thirty-nine mild OSA patients and 25 controls were included (n=64)	OSA	Polysomnography, FOSQ, PVT	There was a statistically significant difference between groups when comparing the “number of lapses” in PVT	Small sample, pilot study
Lancee et al.^20^	Participants with insomnia were divided in two groups: training group (n=67, 49 female and 18 male) and placebo training group (n=70, 51 female and 19 male).	Insomnia	ISI, DT, PSQI	Scores of attentional biases didn’t change at all, the control group showed the same results as the main group, and the study wasn’t able to tell the difference	Online application of DT could be a central factor in the results, conclusion doesn’t show if the attention bias can be altered through this method
Zhao et al.^25^	28 male and 36 female patients diagnosed with insomnia in Shanghai (n=64)	Insomnia and Sleep Disturbance	PSQI, ANT	Scores of alerting efficiencies increased more, while scores of total reaction time decreased more in treatment group, insomnia was found to be associated with higher scores in the executive control variable of the ANT	Small sample
Richards et al.^15^	Older adults, aged 55-89 with Mild Cognitive Impairment and Obstructive Sleep Apnea, divided into two groups: who used CPAP (experimental group, n=29) and those who did not use CPAP (control group, n=25)	Mild cognitive impairment (MCI), OSA	Polysomnography, SCW, PVT, ESS	There were significant decreases in daytime sleepiness, and improvement in attention performance in the two measurements performed, from baseline to six months, and from baseline to one year in the group that adhered to CPAP use compared to the group that did not use CPAP.	Study structure found the groups may have been different on unidentified variables that may have affected the study outcomes, like color/race
Alikhani et al.^23^	46 males with HIV infection (n=46)	Sleep disturbances self-reported	PSQI, ISI, ESS, D2	Treating sleep disturbances over a period of six to 12 weeks had a positive impact on aspects of sleep disturbance, symptoms of depression and anxiety, and cognitive performance	Small sample

OSA: obstructive sleep apnea.

## RESULTS

Among the 15 studies included in this review, it was found that the most common reasons for exclusion at this stage were studies of children and adolescents, mainly with Attention Deficit Hyperactivity Disorder (ADHD) (343 studies). These studies are not required to assess the cognitive part of attention because most of them have treated previously diagnosed ADHD. In this way, the disturbance found in attentional performance will influence sleep disorder, and not the other way around. Thus, there is a tendency to study the consequences of ADHD on healthy sleep, but not to do attentional testing. Another common reason for exclusion was pharmacological studies that investigated a specific drug, or the effect of a drug on patients with sleep disorders, but attention tests were not included (359 studies).

**Figure 1.**
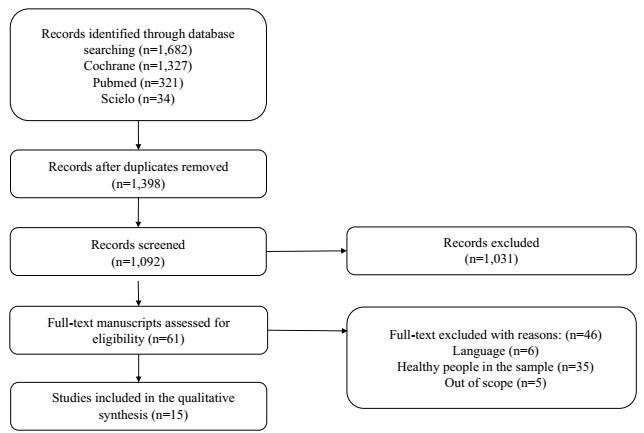
Flowchart of the review process and study selection.

Studies selected from all databases for full-text review were subjected to duplication checking, which resulted in the exclusion of 284 studies. Subsequently, another 348 papers were excluded for not containing relevant data for the present study, such as, for example, inducing sleep deprivation in healthy patients. After the entire process of selecting and applying the eligibility criteria, 15 manuscripts were included in this systematic review. The whole process of selection and application of eligibility criteria is shown in the flowchart of the PRISMA Statement in Figure 1.

Table 1 provides general information about the 15 studies included in this review, ordered by date of publication. The samples were from seven different nationalities: 4 Brazilian, 3 American, 3 Chinese, 1 Dutch, 1 Canadian, 1 Iranian, 1 Swedish, and 1 Italian. The studies were uniformly published between 2011 and 2020, and the 15 publications have a cross-sectional and/or longitudinal design.

The sleep disorders found were: insomnia (4), obstructive sleep apnea (OSA) (6), sleep deprivation (SP) (3), idiopathic REM sleep behavior disorder (iRBD) (1), and narcolepsy (1). Based on the samples, parameters such as the variety of sleep pathologies, the selection of tests for application, the duration of the intervention, and the sample size were analyzed.

Of the six studies that analyzed obstructive sleep apnea, only three performed polysomnographic testing, and, in addition, three others performed the Mini Mental State Examination (MMSE), a brief 30-item questionnaire used to screen for cognitive impairment. In three of these studies, Stroop Color Word Tests (SCWT) were also administered to assess selective attention, ability to maintain focus on an activity, and speed of information processing. Four articles used the Psychomotor Vigilance Test (PVT), a task that involves sustained attention and reaction time and measures the speed with which individuals respond to a visual stimulus. Five of the fifteen articles found used the Pittsburgh Sleep Quality Inventory (PSQI), which refers to sleep quality in the past month, and provides an index of severity and nature of disorder. Seven used the Epworth Sleepiness Scale (ESS), which is a more specific assessment of sleepiness, and ranges from 0 to 24, with excessive sleepiness characterized for scores above 10.

As can be seen from Table 1, the studies do not indicate a strong bias toward the use of any specific test of attention; they simply show that they test more sustained and selective attention. It was also not possible to determine a mean or mode of the attention test results, since it comes from different sleep disorders and this correlation would not be reliable or strong. However, it should be noted that 40% of the studies used the Pittsburgh scale to assess sleep quality, and of the six articles that used PSQI, five of them assessed sustained attention.

## DISCUSSION

Sleep disorders are highly heterogeneous in terms of their causative factors, clinical presentation, and morbidity, making knowledge of the neurological aspects of sleep and the most common sleep disorders essential for a better understanding of the phenomenon. The presence of a sleep disorder correlates with decreased alertness and difficulty in maintaining focus^8^.

This systematic review, therefore, sought to analyze the relationship between sleep disturbance and attentional performance and which type of attention is more impacted or not according to the results of this study. In its most general form, attention can be described as a general level of alertness or the ability to engage with the environment. Likewise, this study focused on the types of attention most commonly described in neuropsychology, namely selective attention, sustained attention, divided attention, and alternate attention. The selected articles sought to answer how sleep disorders such as insomnia, obstructive sleep apnea, narcolepsy, and others impact some of these types of attention.

Among the fifteen studies found, there seems to be a trend toward tests related to sustained attention. Seven studies examined only sustained attention (46.6%), and two evaluated only selective attention (13.3%). Five papers evaluated both types of attention (33.3%), one of the manuscripts analyzed divided attention and selective attention (6.6%), and none of the papers evaluated alternating attention. A Brazilian study addressed executive functions and used the term ‘executive attention’ to refer to a mechanism of attention called Supervised Attentional System (SAS), which deals with inhibitory control of automatic responses as in the Norman and Shallice’s (1986) model^9^. In the present study, we did not consider this concept of attention because it is still discussed by most authors as an executive function skill rather than an attentional skill. It is important to mention that the study of Oliveira et al. (2013) used a type of attention test that can include divided attention, as it is described as “complex attention” or attention as an assessable unit (Table 2)^10^.

**Table 2.** Information about the type of attention and intervention observed in the included studies.

Study	Design	Sample (n)	Nation	Attention type	Intervention?
Bawden et al.^17^	Experimental/Clinical Trial	37	Brazil	Sustained	Yes
Waggoner et al.^12^	Experimental/Randomized Controlled Trial	29	USA	Sustained	No
Moraes et al.^19^	Prospective case-control	19	Brazil	Selective	No
Karimi et al.^16^	Experimental/Clinical Trial	101	Sweden	Selective, Sustained	Yes
Niu et al.^13^	Experimental/Randomized Controlled Trial	62	China	Sustained	Yes
Oliveira et al.^10^	Descriptive and observational	102	Brazil	Selective, “Complex Attention”	No
Castronovo et al.^24^	Experimental/Clinical Trial	32	Italy	Selective, Sustained	Yes
Fortier-Brochu et al.^26^	Prospective case-control	25	Canada	Sustained	No
Djonlagic et al.^14^	Experimental/Controlled Clinical Trial	44	USA	Sustained	Yes
Li et al.^11^	Prospective case-control	46	China	Selective, Sustained	No
Luz et al.^18^	Prospective case-control	64	Brazil	Sustained	No
Lancee et al.^20^	Experimental/Randomized Controlled Trial	137	Netherlands	Selective	Yes
Zhao et al.^25^	Experimental/Randomized Controlled Trial	64	China	Selective, Sustained	Yes
Richards et al.^15^	Experimental/Clinical Trial	25	USA	Selective, Sustained	Yes
Alikhani et al.^23^	Experimental/Randomized Controlled Trial	46	Iran	Sustained	Yes

Among the studies that addressed sleep disorders and tested sustained attention, there was no preference in the tests chosen for evaluation. Of the twelve studies, four used the Psychomotor Vigilance Test (PVT), three used the Mini Mental State Examination (MMSE), which is only a cognitive screening test, two used the Attention Network Test (ANT) and two used the D2 Attention Test. There was also a study by Li et al. (2016)^11^ that evaluated sustained attention using the Dot-Probe Test (DT) to assess the applicability of this test in an online context, which may be a trend for future studies as these tools are needed in times of social isolation and COVID-19.

It is interesting to note that the impact of sleep disorders on sustained attention is notorious. In the study by Waggoner et al.^12^, which observed changes in the nighttime habits of police officers, the PVT test demonstrated twice as many lapses of attention in sleep-deprived individuals compared to the control group. This study contrasts interestingly with two other studies in this review, by Niu and Oliveira, which evaluated the caregivers (in China and Brazil, respectively). The results of the sustained attention tests show an average deficit of 25% in the mean score of the Stroop Color Word Test (SCWT) and 14.8% in the Digit Symbol Substitution Test (DSST)^10^ ^,^ ^11^ ^,^ ^12^ ^,^ ^13^.

Six of the fifteen studies selected in this review addressed OSA and all evaluated sustained attention, providing categorical results. Four of these studies performed some type of intervention, such as double-blind placebo-controlled trials and controlled clinical trials. An interesting contrast is provided by two studies, both conducted in the United States by Djonlagic and Richards, who evaluated the Continuous Positive Airway Pressure (CPAP) treatment, one immediately after the first night of intervention and the second study with follow-ups of 6 months and 1 year after CPAP intervention^14^ ^,^ ^15^. Both studies provided robust results on cognitive assessment after the use of CPAP. Djonlagic’s study concluded that OSA patients treated with CPAP on the first night showed immediate improvement in attentional and vigilance performance the next morning based on PVT results, and OSA patients using CPAP in a long-term treatment showed improvement in sleep quality^14^.

A study by Swedish researchers investigated the prevalence of sleep disorders in Public Transport Operators (PTOs) and evaluated the effects of interventions on hypersomnolence and neurocognitive function in individuals diagnosed with OSA^16^. This study had a satisfactory sample size, performed several examinations (polysomnography, electrocardiography, ANT, CTT, sleep scales, among others), and produced very promising and complete results. Patients diagnosed with an occupational injury during the OSA intervention (59%) were more likely to have an occupational injury than patients without OSA (37%, p<0.08). This study was also based on an intervention for recent diagnoses and demonstrated significant improvements in neurocognitive measures of attention assessed with the Compensatory Tracking Task (CTT)^17^.

It is also noteworthy that four Brazilian studies are included in this review. Two of these studies worked with OSA, one with narcolepsy, and one with sleep deprivation^10^ ^,^ ^18^ ^,^ ^19^ ^,^ ^20^. Interestingly, none of the Brazilian studies had an intervention, and with the exception of Oliveira’s study (n=102), the samples were relatively small (average=40). The study by Moraes was the only one that addressed narcolepsy and assessed attention tests^20^. It is important to mention that this study dealt with executive functions, and the test chosen to assess “executive attention” was the Victoria Stroop Test (VST), which may be a way to assess this Executive Function (EF)^20^. However, the study found that the narcolepsy patients performed significantly slower on the tests than the control group and took up to twice as long to perform the VST and TMT-B.

A Dutch study also stands out because its intervention was very different from other studies^21^. Interestingly, this study evaluated insomnia from a therapeutic perspective, using a randomized, double-blind, placebo-controlled trial design to examine the efficacy of attentional bias modification (ABM) training in treating insomnia as an alternative to Cognitive Behavioral Therapy for Insomnia (CBT-I), which is widely used to treat this sleep disorder.

The Dot-Probe paradigm is a computer-based fast reaction time task in which two clues appear simultaneously in different locations on a computer screen to assess selective attention. The study showed that the ABM approach was not appropriate for treating patients with insomnia and that there were insufficient differences between the ABM-treated patients and the control group, even on tests of selective attention. Studies such as these were rare in this systematic review, once the inclusion criteria were attention tests and randomized trials, and the results showed that there were few such productions.

It is also interesting to mention that all the studies found in this review show impaired attention in sleep disorders, leading to negative effects on daily life and work. In this study, it was not possible to properly identify which type of sleep disorder has a greater impact on selective and sustained attention because of the few number of studies.

However, recent studies have shown that total sleep deprivation for only 24 hours already potentially decreases selective and sustained attention, components of attention, as well as cognitive inhibition, a component of executive functions^22^. Experiments by Norton (1970) with a group of college students indicated that selective attention was the most impacted in sleep-deprived individuals^23^.

In this review, some authors concluded that attention and episodic memory were the most affected aspects. Thus, changes in a more basic physiological phenomenon such as attention were considered additional factors in the global decline in cognitive performance^18^. In turn, Karimi’s study showed that OSA treatment improved drivers’ ability to maintain alertness and performance in standardized situations involving monotonous tasks, as well as their selective attention and episodic memory.

The study by Niu and colleagues showed significant results in groups of nurses who performed tests of attention, indicating that among the types of attention examined in this study, selective attention was the most impaired, severely affecting professional practice and prompting organizations to pay more attention to quality sleep education in this work setting^13^. Although this study does not discuss why sleep affects this type of attention, the results indicate that the loss of precision in tasks involving complex visual information may result from a decrease in sensitivity or a change in bias response.

Confirming the relationship between the impact of sleep disorders on attention, Zhao argues that insomnia triggers a variety of negative emotions, including anxious somatic arousal, rumination, and worry. These negative emotions, in turn, in addition to insomnia itself, may directly lead to a crucial decline in attentional control. Negative emotions was not discussed in the other included manuscripts, which encourages future studies to investigate the correlation between attentional control and emotional comorbidities resulting from sleep disorders^24^ ^,^ ^25^ ^,^ ^26^.

Also in Zhao’s study, it was discussed that a potential mechanism for the impairment of executive attentional control often observed in patients with insomnia could be insufficient slow-wave sleep (SWS) during nighttime sleep. From the perspective of neurological functioning, a study published in Nature in 2017^27^ assessing individuals by examining EEG and local field potentials (LFPs) showed that sleep deprivation alters the functioning of neurons that generate slow and theta waves during wakefulness. Thus, attention and memory lapses are observed during the performance of activities.

In the full reading of the manuscripts, the degree of cognitive impairment (mild, moderate, or severe) was not discussed. What is known is that sleep disorders affect cognition, but vary widely in terms of the cognitive aspect, the disturbance, and the severity of the impact.

Of the seven nationalities examined in the studies, the Brazilian results corroborate the findings of foreign studies, that is, there is a strong correlation between attentional variations and sleep disorders.

However, it is necessary to conduct further studies with specific samples to better inform future research, considering the uniqueness of each type of sleep disorder.

Analysis of the articles selected for this systematic review revealed that there is a significant relationship between sleep and attention performance. In general, the sleep disorders evaluated in this study are related to greater impacts on sustained attention than the other types of attention, but selective attention is also severely compromised.

Despite the strong influence of poor quality of sleep and its impact on attention, the number of studies on sleep and its correlation with attentional performance is small. Nationally, few studies correlated these two topics and undertook interventions to measure their outcomes. Furthermore, most of these the studies are of small sample sizes, making generalizations to the entire population difficult.

Considering that sleep can affect behavior, emotions, and physical and cognitive development, as well as attention levels and other cognitive aspects, it is worth noting that poor sleep habits that may have started in childhood and adolescence can accompany the person in adulthood and lead to sleep deprivation, resulting in more complex problems. These diseases can occur at all stages of development, so further research on the relationship between sleep and attention is needed for scientific progress.
